# Trend in Infective Endocarditis in Bulgaria: Characteristics and Outcome, 17-Years, Single Center Experience

**DOI:** 10.3390/microorganisms12081631

**Published:** 2024-08-09

**Authors:** Bistra Dobreva-Yatseva, Fedya Nikolov, Ralitsa Raycheva, Petar Uchikov, Mariya Tokmakova

**Affiliations:** 1 Section of Cardiology Cardiology Clinic, First Department of Internal Medicine, Faculty of Medicine, Medical University-Plovdiv, UMBAL “St. Georgi” EAD, 4000 Plovdiv, Bulgaria; fedyani@yahoo.com (F.N.); m14021971@yahoo.co.uk (M.T.); 2 Department of Social Medicine and Public Health, Faculty of Public Health, Medical University-Plovdiv, 4000 Plovdiv, Bulgaria; dirdriem@gmail.com; 3 Department of Special Surgery, Faculty of Medicine, Medical University of Plovdiv, 4000 Plovdiv, Bulgaria; petar.uchikov@mu-plovdiv.bg

**Keywords:** infective endocarditis, predisposing heart condition, entry door, comorbidities, complications

## Abstract

Background: Infective endocarditis (IE) remains a difficult disease to diagnose and treat, with a persistently high mortality rate. There is a lack of recent data on IE in Bulgaria over the last decades. Methods: This study is retrospective, single-centered, and includes 270 patients diagnosed with IE for the period 2005–2021. We compared two periods, 2005–2015 (n = 119) and 2016–2021 (n = 151), to find the characteristics changes. Results: The study included 177 (65.5%) male patients. In the second period, there is a significant increase in age from 62 (44–73) to 67 (53–75), (*p* = 0.023); in the Charlson comorbidities index (CCI) from 3 (1–4) to 4 (2–6), (*p* = 0.000); in cases with chronic kidney diseases (CKDs) from 15 (12.6%) to 55 (36.9%), (*p* = 0.001); coronary arterial diseases (CADs) from 20 (16.85%) to 44 (29.1%), (*p* = 0.018); and atrial fibrillation (AF) from 13 (10.9%) to 36 (23.8%), (*p* = 0.006). Ejection fraction decreased significantly in the second period from 63 (56–70) to 59 (51–66), (*p* = 0.000). Almost half of the patients 123 (45.6%) had no known predisposing cardiac condition, and 125 (46.3%) had an unknown port of entry. IE was community-acquired in 174 (64.4%), healthcare-associated in 72 (26.7%), and injection-drug-use-related IE in 24 (8.9%). The study population included 183 (67.8%) native valve IE, 85 (31.5%) prosthetic IE, and 2 (0.74%) intracardiac-device-related IE. The hemocultures were positive in 159 (59.6%), and the most frequent pathogenic agent was staphylococci—89 (33.3%) (*Staphylococcus aureus*—44 (16.5%) and coagulase negative staphylococci—45 (16.8%)). Only 54 (20%) of patients underwent early surgery. The all-cause 30-day mortality rate was 67 (24.8%). There is no significant difference between the two periods in terms of the characteristics listed above. Conclusions: The profile of IE in Bulgaria has changed with increasing age and comorbidity, changing predisposing cardiac conditions, and entry door. The most common pathogen was the *Staphylococcus* spp. The 30-day mortality rate remains high.

## 1. Introduction

Infective endocarditis (IE) was first described more than 4 centuries ago, but it remains a major challenge for physicians. Sir William Osler called it “malignant endocarditis” in a lecture to the Royal College of Physicians in London in 1885 [1,2]. Even then, he noted that few diseases present so many difficulties in diagnosis as malignant endocarditis, and that some of these difficulties are practically insurmountable.

IE is a changing disease in which, despite modern imaging and microbiological techniques, there are often serious difficulties and delays in diagnosis [3]. Improvements in medical and surgical treatment in recent decades have not altered the mortality and serious complication rates. The mortality remains high, up to 30% [4,5]. The challenges associated with infective endocarditis are greater than ever due to the changing profile of the disease, both on the patient side and on the microbiological pathogen side. Characteristics of IE change over time and depend on the geographical and socioeconomic level of the country. In recent decades there has been an increase in the patients’ age, comorbidity, staphylococcal etiology, and healthcare-related IE. There is a shift in predisposing factors with an increase in cases of degenerative valve disease, prosthetic valves, indwelling catheters and implanted cardiac devices, and intravenous drug users, while cases of rheumatic heart disease are becoming less common in developed countries [4,5,6].

Knowing the current profile of patients with IE helps to ensure timely and accurate diagnosis, which is key to initiating appropriate treatment. This is associated with a reduction in in-hospital mortality and an improvement in the long-term prognosis of patients [7,8,9].

There are no data for Bulgaria for the last few decades. Up-to-date IE data for Europe are available from EURO–ENDO, but Bulgaria is not included in this register. We aim to describe the characteristics of IE and its changes over the 17 years period.

## 2. Material and Method

This study is retrospective, single-centered, including 270 patients with a diagnosis of IE, according to the modified Duke criteria, treated at the University Hospital “St. Georgi”, in the city of Plovdiv for the January 2005–December 2021 period. We compared two periods, 2005–2015 (n = 119) and 2016–2021 (n = 151), to find the characteristics changes. The hospital capacity is 1500 beds, and the cardiology clinic is a reference center for the treatment of IE for a large part of southern Bulgaria. The medical records of treated patients with codes I33, I38, and I39 for the described period were used. Variables studied included demographics, risk group, presence of predisposing heart disease, comorbidities, Charlson comorbidity index (CCI) [10], entry gate, predictors for transient bacteremia, clinical, echocardiographic findings, causative organisms, complications, and clinical outcome.

## 3. Definition and Classification of IE

The diagnosis was defined as definite IE or possible IE according to the modified Duke criteria [11]. Surgical treatment of IE was defined as early when the surgery was performed during antibiotic treatment. Valvular involvement of IE is determined based on findings from echocardiography, other imaging studies, cardiac surgery, or in some cases by clinical presentation. Episodes of IE were categorized by mode of acquisition as: community-acquired IE (CAIE), healthcare-associated IE (HAIE), and intravenous-drug-use-associated IE (IDUIE). These categories are mutually exclusive. IE was defined as HAIE according to the following criteria: (1) occurrence of IE > 48 h after hospital admission or within 6 months after hospital discharge for ≥ 2 days; (2) IE developed within 6 months after a significant invasive procedure performed during hospitalization or in an outpatient setting; (3) extensive outpatient healthcare contacts, defined as receiving wound care or intravenous treatment within 1 month before the onset of IE; or (4) stay in a clinic-home to receive similar care [12,13,14,15]. IE occurring on a prosthetic valve within 12 months of surgery is defined as prosthetic valve early endocarditis (PVIE) and is classified as HAIE. Patients with a recent (within 1 month) or longer history of intravenous drug use were classified as IDUIE. Patients with no medical history and no history of injecting drug use were classified as CAIE. IE following dental treatment is considered to be CAIE if there is no other healthcare contact. The presence of septic emboli and an extracardiac focus of infection was defined as a focus of infection detected by imaging or based on typical clinical presentation. Complications were diagnosed according to the established diagnostic criteria and recommendations.

## 4. Statistical Methods

Quantitative data are presented as arithmetic mean ± standard deviation (mean ± SD) or median and interquartile range (25–75 percentiles) according to the type of distribution of the variables (Kolmogorov–Smirnov test). Categorical variables were summarized using absolute (n) and relative (%) magnitudes. A Mann–Whitney test for independent samples was used to compare quantitative variables between two groups. A z-test was used to compare the relative shares of categorical variables between the studied groups. A *p*-value < 0.05 (two-tailed test) was considered statistically significant for all tests. A statistical analysis was performed using SPSS, version 26.0 (IBM Corp., Binghamton, NY, USA).

## 5. Results

Of all 270 patients, 205 (75.9%) had definite IE, with 133 (65%) of them having two major criteria and 72 (35%) having one major and three minor criteria. There were 65 (24.1%) diagnosed with possible IE, 62 (95%) of them with one major and one minor criterion and three with three minor criteria. The patients’ baseline characteristics are shown in Table 1. The median age was 65 (51–74), and the patients were significantly older in the second time period (67 (53–75) vs. 62 (44–73), *p* = 0.023). We found an increasing number of cases per year in the second period. The median time from symptom onset to hospitalization was 30 (20–60) days, with no difference between the two periods. Almost half of the patients, 136 (50.4%), are at low risk; 90 (33%) are at high risk and should receive IE prophylaxis. Native valve IE was prevalent, 180 (66.7%); prosthetic IE was 88 (32.6%); and CDRIE was 2 (0.74%). The entry door was unknown in almost half of the cases, 122 (46.3%), and the most common gateway was non-dental manipulation/procedures, 44 (16.3%), including cardiovascular interventions, skin procedures and wound management, transfusion, bone marrow puncture, and endoscopic procedures. The next in terms of frequency are dental procedures, 30 (11.1%); intravenous drug users, 24 (8.9%); and hemodialysis, 13 (4.8%). We found an increase in cases with non-dental manipulations/procedures and hemodialysis in the second period, without significant differences. The most common cardiac predisposition was prosthetic heart valves 76 (28.2%) and almost half of the patients 123 (45.6%) had no known cardiac disease. According to the mode of acquisition, we found community-acquired IE in 174 (64.4%), healthcare-related IE in 72 (26.7%), and intravenous-drug-use-related IE in 24 (8.9%), with no significant difference between the two periods.

We found a wide range of comorbidities and a significant increase in CCI in the second period from 3 (1, 4) to 4 (2, 6), *p* = 0.000 (Table 2). The most common comorbidities were arterial hypertension, 171 (63.3%); chronic heart failure, 124 (45.9%); previous cardiac surgery, 95 (35.2%); chronic kidney disease (CKD), 70 (25.9%); coronary artery disease (CAD), 64 (23.7%); diabetes, 51 (18.9%); and atrial fibrillation (AF), 49 (18.1%). We reported significant increases in the second period for CKD from 15 (12.6%) to 55 (36.9%), *p* = 0.001; CAD from 20 (16.8%) to 44 (29.1%), *p* = 0.018; and AF from 13 (10.9%) to 36 (23.8%), *p* = 0.006 (Table 2).

The most common clinical presentations were fever, 263 (97.4%); anemia, 248 (92.5%); heart murmur, 178 (66.2%); splenomegaly, 49 (18.1%); and skin disorders, 14 (5.5%). We found a significant increase in cases with anemia in the second period (from 104 (88.9%) to 144 (95.4%), *p* = 0.044).

The 30-day mortality rate was 67 (24.8%) patients, with no significant change between periods. Early surgery was performed in 54 (20%), rising from 20 (16.8%) to 34 (22.5%) in the second period, with no significant difference. The most common complications were acute heart failure, 128 (47.5%); worsening kidney function, 111 (41.1%); embolism, 56 (20.7%); stroke, 30 (11.1%); and septic shock, 23 (8.5%) (Table 2).

Transthoracic echocardiography was performed in 100% of patients, and transesophageal echocardiography was performed in 97 (35.9%) of them (Table 3). We found vegetation in 226 (83.7%), paravalvular abscess in 8 (3%), chordal rupture in 5 (3.3%), and valve obstruction in 32 (11.9%). The distribution of valvular regurgitation according to severity was as follows: mild/moderate: AV—79 (29.3%); MV—64 (23.7%); TV—16 (6%). Severe regurgitation: AV—67 (24.8%); MV—51 (19.3%); TV—20 (7.4%).

Single-valve IE (SIE) was found in 223 (82.6%), multivalvular IE (MIE) in 45 (16.66%), and CDRIE in 2 (0.74%). The most frequently affected valve was aortic (AV), 121 (44.8%); followed by mitral valve, (MV) 75 (27.8%); and tricuspid valve (TV), 26 (9.62%). We had one case (0.37%) with pulmonary valve IE. Of the MIE, the most common was AV–MV IE—37 (13.7%) (Table 3). There was no triple- or quadruple-valve endocarditis in our series. We found vegetation in 226 (83.7%) and significantly increased the cases with vegetation 10–15 mm in the second period (from 7 (5.9%) to 31 (20.5%), *p* = 0.001 *. We also found a significant decrease in EF in the second period from 63% (56–70) to 59% (51–66), (*p* = 0.000 †).

We had 111 (41.1%) negative blood cultures, and the most common pathogens were staphylococci 89 (33%)—*Staphylococcus aureus* 44 (16.3%) and *Staphylococcus CoNS* 45 (16.7%). We found enterococci in 25 (9.3%), streptococci in 21 (7.7%), Gram-negative, non-HASEK in 19 (7.0%), with no difference between the two periods. Only other *Streptococci* decreased significantly in the second period from 4 (3.4%) to 0 (0%), *p* = 0.022 (Table 4).

## 6. Discussion

The average age of patients with IE has increased significantly in recent decades. In our study, the median age of patients was 65 years. In the subgroup analysis, a statistically significant increase in age was found in the second period, 67 years compared to 62 years, over a period of 6 years. Recent data from other economically developed countries are similar: EURO–ENDO mean age was 59.25 ± 18.03 years [16]; France, 69 years [6]; Japan, 69.1 years [17]; Canada, 56 years [18]; Spain, 61.8 years [19]; Portugal, 68.3 years [20]; Netherlands, 67.5 years [21]; and South Korea, 56 years [22]. A number of factors have contributed to this changing age distribution in countries with a high standard of living. The predisposing cardiac risk factors in many of these countries have shifted from rheumatic valvular disease, which occurs predominantly in young patients, to degenerative valvular disease, which occurs predominantly in the elderly. Age remains lower in less economically developed countries, where the dominant predisposing factors remain rheumatic heart disease (RHD) and congenital heart disease and the increase in intravenous drug addiction. This is illustrated by an 11-year study in India, where the average age of patients was 34.1 years, with predisposing factors of RHD (20%) and i.v. drug dependence of 30% [23]. Similar data are available for Iran, 39.7 years [24]; Vietnam, 37.6 years [25]; and Pakistan, 46.9 years [26]. Age is an important characteristic, as it is associated with increased comorbidity, a greater proportion of patients with IE, healthcare-related difficulties, treatment difficulties, and a greater proportion of patients with an unfavorable outcome.

In the total study sample, 65.6% were men. Data from other studies from different time periods and geographical locations are similar. The male predominance, about 2/3 of IE cases, has not changed historically. The gender distribution is also independent of the geographical and socio-economic status of the countries.

The distribution of patients by risk groups in our study is comparable to other studies. The high-risk group included patients with prosthetic valve IE (PVIE), patients with past IE, and a very low percentage of cyanotic uncorrected congenital heart diseases. In our sample, the majority were PVIE (28.2%) and experienced IE (7.4%), with 5.2% having experienced prosthetic valve IE. In comparison, the high-risk group in EURO–ENDO is 37% (5); India, 45.6% (23); Africa, 29.1% [27]; and Portugal, 38% [28]. The latest recommendations of the ESC (9) recommend IE prophylaxis only for high-risk groups. The reduction in the number of cases with a dental entry door and the reduction in the proportion of streptococci as causative agents worldwide are arguments in favor of restricting IE prophylaxis. In our study, 2/3 of patients with IE did not receive prophylaxis. These data are likely to be relevant to further discussion of the effect of restrictions on the prevention of IE.

Predisposing cardiac conditions are an important part of the pathogenesis of IE. Their spectrum and distribution have changed considerably in recent decades, with significant differences in geographical and socioeconomic status between countries. In the past, the most common predisposing conditions were rheumatic heart disease and congenital heart disorders, which continue to be the most common in under- and medium-developed countries [24,25,26]. In our results, the largest proportion of patients did not have a history of valve disease. It has been shown that 30–40% of degenerative valve lesions are of unknown etiology [29]. Based on our data, the most frequent predisposing factor is the presence of a prosthetic heart valve in almost one-third of patients. These data are similar to those in European and other economically developed countries, where PVIE cases are increasing. In comparison, PVIE cases in ICE–PCS were 21% [4]; France, 25% [6]; Euro Heart Survey, 26% [30]; EURO–ENDO, 30% [5].

The port of entry was unknown in almost half of patients in our study. Data from a study in Romania are similar—34% [31]. Donova found an unknown port of entry in 68.6% [32]. This was followed by manipulation/procedure, dental, i.v. drug addiction, and hemodialysis. The remaining categories—skin, genitourinary, gastrointestinal, respiratory, ear–nose–throat, and others—are represented by less than 5%. Data from EURO–ENDO are similar, with gastrointestinal gateways accounting for 6.3%; urogenital, 4.5%; and i.v. drug dependence, 6.9%. The dental route is of particular interest in the context of IE prevention restrictions. A high proportion of patients with a dental portal was reported in a meta-analysis for Africa (28.3%), with an underlying predisposing cardiac condition of rheumatic heart disease [27]. Our data are close to those of EURO–ENDO-9.8%, where they reported a decrease in cases with a dental portal. For comparison, in the Euro Heart Survey, it is 15% [30]; French Registry, 20.6% [6]; and ICE–PCS, 17% [4]. These data correlate with a decrease in the proportion of streptococci as the causative agent. Based on our data, manipulation/procedure was the largest proportion of entry doors. This reflects the global increase in staphylococci and enterococci as causative agents and the increasing proportion of healthcare-associated IE.

The most common clinical symptoms in our study were fever, anemia, and heart murmur. We reported a statistically significant increase in the proportion of anemia in G2 compared to G1 (*p* = 0.0436 *). This led to a statistically significant increase in CKD patients in G2. Splenomegaly and skin changes were found in small percentages. We observed a decrease in cases with splenomegaly and skin changes in G2 compared to G1, without statistical significance. Our data are comparable to those from EURO–ENDO and other studies from the last five years. Most patients in recent decades have few of the classic clinical findings traditionally associated with IE, which is a modern trend. For example, in the 1960s and 1970s, 11–23% of patients with IE had Osler’s nodes, and 20–44% had splenomegaly [8]. In recent decades, a significant decrease in cases with typical skin changes (immunological and embolic) has been observed. In another study from 2014 [33], Servy et al. found skin changes in 11.9% of 497 patients. These were purpura—8%; Osler’s nodes—2.7%; Janeway lesions—1.6%; and conjunctival hemorrhages—0.6%.

In our sample, TEE was performed in 35.9% of patients. The data for Canada are similar—29.4% [18]. TEE was performed more frequently in Japan—73.3% [17]; Latin America—59.6% [27]; ICE–PCS—59% [4]; EURO–ENDO—58.1% [5]; Iran—54.4% [24]. TEE was performed significantly less frequently in India—18.1% [23]. However, we found a high percentage of vegetations—83.7%. TEE is known to be difficult to perform in patients with severe or critical illness. TEE is the gold standard in the diagnosis of IE, especially in cases of PVIE and CDRIE, and its wider use is recommended.

We found valvular vegetations in 83.7%, and this result is comparable with other studies. According to our data, the most frequently affected valve is the aortic valve. Data from EURO–ENDO, Latin America, and Canada are similar. In the remaining studies, mitral valve involvement is the most common. High percentages of tricuspid valve involvement were found in India—30.2%; Iran—20.7%; and Canada—15%. This is due to the widespread use of intravenous drug addiction as a predisposing factor in these countries.

Regarding the size of valve vegetations, the highest proportion is found in those smaller than 10 mm, 56.7%, and in the subgroup analysis, we find a statistically significant increase in vegetations with a size of 10–5 mm in G2 (20.5%) compared to G1 (5.9%). This is associated with an increase in cases of *staphylococcal* IE. Correspondingly, vegetations smaller than 10 mm decreased significantly in G2 compared to G1. A higher proportion of vegetations over 10 mm was found in Canada, 44.6%; India—10–30 mm, 38.7% and over 30 mm, 21.6%; Romania—over 10 mm, 42.8%; Vietnam—10–15 mm, 31.2% and over 15 mm, 7.4%. Vegetations greater than 10 mm are associated with a higher risk of embolism and are an indication for early surgical intervention to prevent embolism [9].

A statistically significant difference was reported in ejection fraction (EF) in the subgroup analysis, with a lower EF for G2 compared to G1. This was the result of a significantly higher proportion of patients with CAD and atrial fibrillation, as well as the higher CCI in G2.

The distribution of severity of regurgitation was similar to other studies. We found a perivalvular abscess and chordal rupture in low percentage. The incidence of this complications varies between studies [4,5,23,27,34,35].

The microbiological results in our sample are comparable with changes and trends over the last decades. The leading cause of IE are staphylococci, with a decrease in the proportion of streptococci and an increase in the proportion of enterococci and Gram-negative (GNB) micro-organisms. This is directly related to the increase in the percentage of healthcare-associated IE, intravenous-drug-associated IE, and the decrease in the incidence of dental entry door. These data are comparable to those reported in almost all current studies [4,5,6,27,36]. An exception is the data from South Korea, where the most common causes of IE are streptococci [22].

The proportion of negative blood cultures varies widely (10–52%) and is mostly related to previous long-term antibiotic treatment [34]. The other factors for blood culture negative IE are associated with fastidious slow-growing bacteria, including Coxiella burnetii (causing Q fever), *Bartonella* spp., *Brucella* spp., *Mycoplasma* spp., *Legionella* spp., and *Tropheryma whipplei*; non-bacterial organisms—fungi; and local microbiological resources. Specific serological and polymerase chain reaction (PCR) tests are required for these pathogens and should also be considered. In up to 60% of these cases, the pathogen can be isolated [35]. Our data showed negative blood cultures in 40.4%, mainly due to previous antibiotic treatment. In comparison, a higher proportion of negative blood cultures were found in Iran—56%; Pakistan—54%; Portugal—52%; and Africa—51.4%. The fewest cases of negative blood cultures were observed in Japan—5%; ICE–PCS—10%; Canada—18%; EURO–ENDO—21%; Latin America—24%; and South Korea—26.3%. Our data are close to those of France—35.8% and India—36%.

The current trend worldwide, especially in economically developed countries, is to increase the proportion of HAIE. This is the result of an aging population, improvements in healthcare, technological advances in medicine, and an increase in the average age of IE patients [4,5]. We found no significantly increased incidence of healthcare-associated IE in G2 compared to G1.

In-hospital complications are an important feature of patients with IE and are directly related to the outcome of the disease. The 30-days mortality remains high and unchanged despite advances in diagnosis, including new imaging modalities and treatment with the use of new antibiotic molecules and early surgery. According to our data, in-hospital mortality up to 30 days is 24.8%, which is comparable to data worldwide. Data from studies and meta-analyses with large numbers of patients show an in-hospital mortality rate of around 20–25%. Relatively lower mortality rates are reported by South Korea—14.6%; India—17%; EURO–ENDO—17.1%; and ICE–PCS—18%. For the first two countries, this is probably due to a lower mean age and correspondingly lower patient comorbidity. For the other two registries, the high rate of early surgery is noteworthy, 51.2% and 48%, respectively. The highest in-hospital mortality rate was reported for Iran—34.1% in a relatively small study [24]. Acute heart failure is the most frequent complication, both in our data and in other studies, with the exception of India. There, the average age (34.1 ± 13.7 years) and comorbidity are low. Based on our data the high proportion of patients with impaired kidney function can be explained by a higher proportion of patients with CKD, worsened renal function as a result of antibiotic treatment, and immunological changes, as well as circulatory and systemic disorders in acute heart failure and septic shock. The rates of embolic events and septic shock in our study were similar to those in EURO–ENDO. Acute neurological complications occur in 20–40% of patients with IE [37]. The incidence of acute neurological complications in our study is similar to that in Portugal, Iran, India, and Africa. A significantly higher proportion is found in EURO–ENDO. It is likely that the more frequent use of CT and MRI increases the diagnosis of acute neurological complications.

Early surgery is a protective indicator and failure to perform early surgery when indicated is a strong predictor of in-hospital death [5,17,36]. In our sample, early surgery was performed in 20% of cases in G0, with an increase from 16% to 22.5% in G2 compared with G1, without statistical significance. In comparison, the highest percentage of early surgery was performed in South Korea—65.2% [22]; followed by Iran—57.6% [24]; Romania—51.7% [31]; EURO–ENDO—51.2% [5]; Africa—49.1 [27]; Canada—48% [18]; ICE–PCS 48% [4]; and France—45% [6]. The fewest number of patients treated with early surgery were in India—13.1% [23]; Portugal—13.2% [20]; Japan—17.2% [17]; and Russia—17% [38]. Refractory heart failure, septic shock (persistent infection), and prevention of embolism are established indications for early surgery by ECS (2023) [9].

## 7. Limitation

This study is retrospective, and the data were based on the clinical database of a single center. We did not use the nuclear imaging diagnostics (18F-fluorodeoxyglucose positron emission tomography and leucocyte scintigraphy) due to unavailable resources. This is a very important study and major criteria for diagnosis, especially in cases of prosthetic IE and minor criteria if extracardiac foci of infection are found. In cases of negative blood culture, these data are crucial for diagnosis. Another limitation of our study is that only in-hospital follow-up was available. Despite these limitations, our study is the only one on this subject in Bulgaria for the last decades that includes a large number of patients for a long period of time—17 years.

## 8. Conclusions

The profile of IE in Bulgaria has changed with increasing age and comorbidity, changing the predisposing cardiac conditions and entry door. The most common pathogens were staphylococci. In-hospital mortality remains high.

## Figures and Tables

**Table 1:** Baseline characteristics.

Variables	2005–2021 G0 n = 270	2005–2015 G1 n = 19	2016–2021 G2 n = 151	*p* Value
Age in yrs., Χ ± SD *Median*, *(IQR)*	60.86 ±16.83 65 (51–74)	58.13 ± 17.71 62.0 (44–73)	63.01 ± 15.84 67 (53–75)	0.023 †
Gender, male, n (%)	177 (65.6)	79 (66.4)	98 (64.9)	0.7968 *
Time symptoms–hospita-lization, *Median*, *(IQR)*	30 (20–60)	30 (14–60)	30 (20–60)	0.932 †
Previous AB treatment	142 (52.6)	59 (49.6)	82 (54.3)	0.443 *
Risk groups, n (%)				
Low	136 (50.4)	55 (46.2)	81 (53.6)	0.227 *
Moderate	44 (16.3)	24 (20.2)	20 (13.2)	0.122 *
High	90 (33.3)	40 (33.6)	50 (33.1)	0.931 *
Type of valves, n (%)				
Native IE	180 (66.7)	77 (64.7)	103 (68.2)	0.629 *
Prosthetic IE	88 (32.6)	41 (34.4)	47 (31.1)	0.567 *
Late prosthetic	9 (3.3)	6 (5.0)	3 (2.0)	0.172 *
Early prosthetic	79 (29.3)	35 (29.4)	44 (29.1)	0.957 *
CDRIE	2 (0.7)	1 (0.8)	1 (0.7)	0.061 *
Entry door, n (%)				
Unknown	125 (46.3)	57 (47.9)	68 (45)	0.635 *
Non-dental manipulation/Procedures	44 (16.3)	15 (12.6)	29 (19.2)	0.145 *
Dental Procedures	30 (11.1)	14 (11.8)	16 (10.6)	0.756 *
I.v. drug users	24 (8.9)	13 (10.9)	11 (7.3)	0.302 *
Hemodialysis	13 (4.8)	3 (2.5)	10 (6.6)	0.117 *
Skin	10 (3.7)	2 (1.7)	8 (5.3)	0.120 *
Urogenital	9 (3.3)	3 (2.5)	6 (4.0)	0.496 *
Gastrointestinal	5 (1.9)	5 (4.2)	0 (0)	0.011 *
Respirators	5 (1.9)	5 (4.2)	0 (0)	0.011 *
Ear Nose Throat	4 (1.5)	2 (1.7)	2 (1.3)	0.787 *
Others	1 (0.4)	(0)	1 (0.7)	0.361 *
Predisposing heart conditions, n (%)				
Prosthetic valve	76 (28.2)	33 (27.7)	43 (28.5)	0.899 *
Past IE	20 (7.4)	10 (8.4)	10 (6.6)	0.575 *
Past IE prosthetic	14 (5.2)	7 (5.9)	7(4.6)	0.632 *
Past IE native valves	6 (2.2)	3 (2.5)	3 (2.0)	0.782 *
Rheumatic heart disease	11 (4.0)	7 (5.9)	4 (2.6)	0.172 *
Congenital heart disease	21 (7.8)	10 (8.4)	11 (7.3)	0.738 *
Bicuspid Ao valve	11 (4.1)	5 (4.2)	6 (4.0)	0.934 *
Mitral valve prolapse	8 (3.0)	4 (3.4)	4 (2.6)	0.734 *
Other	2 (0.7)	1(0.8)	1 (0.7)	0.924 *
Degenerative valve	19 (7.0)	8 (6.7)	11 (7.3)	0.848 *
Without	123 (45.6)	51 (42.9)	72 (47.7)	0.432 *
Type of acquisition				
Community-acquired IE	174 (64.4)	76 (63.8)	98 (64.9)	0.747 *
Health-care-associated IE	72 (26.7)	29 (24.4)	43 (28.5)	0.350 *
Intravenous-drug-use-related IE	24 (8.9)	14 (11.8)	10 (6.6)	0.091 *

* *z*-test; † Mann–Whitney U Test; AB—antibiotic; CDRIE—cardiac device related IE.

**Table 2:** Comorbidities, clinical symptoms, and complications.

Variables	2005–2021 G0 n = 270	2005–2015 G1 n = 119	2016–2021 G2 n = 151	*p* Value
Comorbidity				
CCI, *Median, (IQR)*	3 (2–5)	3 (1–4)	4 (2 = 6)	0.000 †
AH	171 (63.3)	69 (58.0)	102 (67.5)	0.108 *
CHF	124 (45.9)	61 (51.3)	63 (41.7)	0.279 *
Heart surgery	95 (35.2)	43 (36.1)	52 (34.4)	0.772 *
CKD	70 (25.9)	15 (12.6)	55 (36.9)	0.001 *
CAD	64 (23.7)	20 (16.8)	44 (29.1)	0.018 *
Diabetes	51 (18.9)	19 (16.0)	32 (21.2)	0.279*
Atrial fibrillation	49 (18.1)	13 (10.9)	36 (23.8)	0.006 *
Past stroke	40 (14.8)	16 (13.4)	24 (15.9)	0.566 *
Gastrointestinal	32 (11.1)	13 (10.9)	19 (12.6)	0.668 *
Malignancy	30 (11.1)	11 (9.2)	19 (12.6)	0.377 *
COPD	21 (7.8)	6 (15.0)	15 (10.0)	0.213 *
Hemodialysis	14 (5.2)	3 (2.5)	11 (7.3)	0.077 *
Chronic liver disease	13 (4.8)	7 (5.9)	6 (4.0)	0.470 *
Systemic disease	4 (1.5)	1 (0.8)	3 (2.0)	0.416 *
Clinical symptoms				
Fever	263 (97.4)	115 (96.6)	148 (98)	0.474 *
Anemia	248 (92.5)	104 (88.9)	144 (95.4)	0.044 *
Cardiac murmur	178 (66.2)	77 (64.7)	101 (67.3)	0.654 *
Splenomegaly	49 (18.1)	25 (21.0)	24 (15.9)	0.280 *
Skin disorders	14 (5.5)	9 (7.6)	5 (3.3)	0.114 *
Complications				
Outcome 30 days-died, n (%)	67 (24.8)	30 (25.2)	37 (24.5)	0.895 *
Early surgery, n (%)	54 (20.0)	20 (16.8)	34 (22.5)	0.245 *
AHF	128 (47.5)	58 (48.7)	70 (46.4)	0.707 *
Septic shock	23 (8.5)	11 (9.2)	12 (7.9)	0.703 *
Strocke	30 (11.1)	11 (9.2)	19 (12.6)	0.377 *
Embolism	56 (20.7)	24 (21.0)	31 (20.5)	0.920 *
Brain	29 (51.7)	11 (45.8)	18 (58)	0.369 *
Lung	5 (8.9)	0 (0.0)	5 (16.1)	-
Spleen	10 (17.9)	4 (16.7)	6 (19.4)	0.800 *
Skin	7 (12.5)	6 (25)	1 (3.22)	0.016 *
Musculoskeletal	2 (3.57)	2 (8.3)	0 (0.0)	-
Combine	3 (5.4)	2 (8.3)	1 (3.2)	0.408 *
Worsening kidney function	111 (41.1)	43 (36.1)	68 (45.0)	0.140 *

* *z*-test; † Mann–Whitney U Test; CCI—Charlson comorbidity index; AH—arterial hypertension; CHF—chronic heart failure; CKD—chronic kidney diseases; CAD—coronary arterial diseases; COPD—chronic obstructive pulmonary diseases; AHF—acute heart failure.

**Table 3:** Echocardiogram findings.

Variables	2005–2021 G0 n = 270	2005–2015 G1 n = 119	2016–2021 G2 n = 151	*p* Value
TTE	270 (100)	119 (100)	151(100)	N/A
TTE + TOE	97 (35.9)	42 (35.3)	55 (36.4)	0.8516 *
Valve location, n (%)				
AV	121 (44.8)	55 (46.2)	66 (43.7)	0.682 *
MV	74 (27.4)	29 (24.4)	45 (29.8)	0.323 *
TV	26 (9.6)	11 (9.2)	15 (9.9)	0.846 *
PV	1 (0.4)	0 (0.0)	1 (0.7)	0.361 *
Bivalve IE	45 (16.7)	23 (19.3)	22 (14.6)	0.304 *
AV–MV	37 (13.7)	20 (16.8)	17 (11.3)	0.192 *
AV–TV	4 (1.5)	1 (0.8)	3 (2.0)	0.416 *
MV–TV	4 (1.5)	2 (1.7)	2 (1.3)	0.787 *
CDRIE	2 (0.74)	1 (0.8)	1 (0.66)	0.693 *
Vegetations, n (%)	226 (83.7)	95 (79.8)	131 (86.8)	0.122 *
<10 mm	153 (56.7)	77 (64.7)	76 (50.3)	0.018 *
10–15 mm	38 (14.1)	7 (5.9)	31 (20.5)	0.001 *
>15 mm	35 (13.0)	11 (9.2)	24 (15.9)	0.104 *
Perivalvular abscess, n (%)	8 (3.0)	3 (2.5)	5 (3.3)	0.670 *
Chordal rupture, n (%)	5 (3.3)	1 (0.7)	4 (2.6)	0.239 *
EF %, *Mediana, (IQR)*	60 (54–68)	63 (56–70)	59 (51–66)	0.000 †
Valve obstruction, n (%)	32 (11.9)	13 (10.9)	19 (12.6)	0.668 *
Aortic regurgitation, n (%)	146 (54)	66 (60.0)	80 (53.0)	0.250 *
Mild–moderate	79 (29.3)	32 (26.8)	44 (29.2)	0.663 *
Severe	67 (24.8)	34 (28.5)	36 (23.9)	0.392 *
Mitral regurgitation, n (%)	115 (42.6)	52 (43.7)	63 (41.7)	0.775 *
Mild–moderate	64 (23.7)	29 (24.3)	35 (23.2)	0.833 *
Severe	51 (18.9)	23 (19.3)	28 (18.5)	0.868 *
Tricuspid regurgitation, n (%)	36 (13.3)	14 (11.8)	22 (14.6)	0.502 *
Mild–moderate	16 (6.0)	6 (5.0)	10 (6.6)	0.579 *
Severe	20 (7.4)	8 (6.8)	12 (7.9)	0.732 *

* *z*-test; † Mann–Whitney U Test; TTE—transthoracic echocardiography; TOE—transesophageal echocardiography; AV—aortic valve; MV—mitral valve; TV—tricuspid valve; CDRIE—cardiac device related IE; EF—injection fraction.

**Table 4:** Microbiological agent.

Microbiological Agent n (%)	2005–2021 G0 n = 270	2005–2015 G1 n = 119	2016–2021 G2 n = 151	*p* Value *
*Negative hemoculture*	111 (41.1)	48 (40.3)	63 (41.8)	0.803
*Staphylococci*	89 (33.0)	39 (32.8)	50 (33.1)	0.958
*Staphylococcus aureus*	44 (16.3)	16 (13.5)	28 (18.5)	0.269
*Staphylococcus CoNS*	45 (16.7)	23 (19.3)	22 (14,6)	0.303
*Streptococci*	21 (7.7)	12 (10.1)	9 (6.0)	0.212
*Streptococcus viridans*	9 (3.4)	6 (5.0)	3 (2.1)	0.190
*Streptococcus beta-hemolyticus*	2 (0.7)	0 (0.0)	2 (1.3)	0.212
*Streptococcus alfa hemolyticus*	6 (2.2)	2 (1.7)	4 (2.6)	0.617
*Streptococci—others*	4 (1.5)	4 (3.4)	0 (0.0)	0.022
*Enterococci*	25 (9.3)	9 (7.6)	16 (10.5)	0.413
*Enterococcus species*	1 (0.4)	0 (0.0)	1 (0.65)	0.378
*Enterococus faecalis*	23 (8.5)	9 (7.6)	14 (9.2)	0.639
*Enterococcus durans*	1 (0.4)	0 (0.0)	1 (0.65)	0.378
*Gram-negative* (non-HACEK)	19 (7.0)	8 (6.7)	11 (7.3)	0.848
*Pseudomonas aeruginosa*	2 (0.7)	0 (0.0)	2 (1.3)	0.212
*Escherichia coli*	9 (3.3)	3 (2.5)	6 (4.0)	0.496
*Enterobacter cloacae*	1 (0.4)	1 (0.8)	0 (0.0)	0.271
*Klebsiella pneumoniae*	3 (1.1)	2 (1.7)	1 (0.65)	0.414
*Serratia marcescens*	4 (1.5)	2 (1.7)	2 (1.3)	0.887
*Others*	5 (1.9)	3 (2.5)	2 (1.3)	0.465
*Candida* spp.	3 (1.1)	3 (2.5)	0 (0.0)	0.051
*Erysipelothix rhusiopathiae*	1 (0.4)	0 (0.0)	1 (0.65)	0.378
*Brevibacterium casei*	1 (0.4)	0 (0.0)	1 (0.65)	0.378

*z*—test *; CoNS—coagulase-negative staphylococcus; non-HACEK—(Hemophilus species, Actinobacillus, Cardiobacterium, Eikenella, or Kingella).

## Data Availability

The original contributions presented in the study are included in the article, further inquiries can be directed to the corresponding author.
